# Parrots Eat Nutritious Foods despite Toxins

**DOI:** 10.1371/journal.pone.0038293

**Published:** 2012-06-05

**Authors:** James D. Gilardi, Catherine A. Toft

**Affiliations:** Department of Evolution and Ecology, University of California Davis, Davis, California, United States of America; University of Lethbridge, Canada

## Abstract

**Background:**

Generalist herbivores are challenged not only by the low nitrogen and high indigestibility of their plant foods, but also by physical and chemical defenses of plants. This study investigated the foods of wild parrots in the Peruvian Amazon and asked whether these foods contain dietary components that are limiting for generalist herbivores (protein, lipids, minerals) and in what quantity; whether parrots chose foods based on nutrient content; and whether parrots avoid plants that are chemically defended.

**Methodology/Principal Findings:**

We made 224 field observations of free-ranging parrots of 17 species in 8 genera foraging on 102 species of trees in an undisturbed tropical rainforest, in two dry seasons (July-August 1992–1993) and one wet season (January-February1994). We performed laboratory analyses of parts of plants eaten and not eaten by parrots and brine shrimp assays of toxicity as a proxy for vertebrates. Parrots ate seeds, fruits, flowers, leaves, bark, and insect larvae, but up to 70% of their diet comprised seeds of many species of tropical trees, in various stages of ripeness. Plant parts eaten by parrots were rich in protein, lipid, and essential minerals, as well as potentially toxic chemicals. Seeds were higher than other plant materials in protein and lipid and lower in fiber. Large macaws of three species ate foods higher in protein and lipids and lower in fiber compared to plant parts available but not eaten. Macaws ate foods that were lower in phenolic compounds than foods they avoided. Nevertheless, foods eaten by macaws contained measurable levels of toxicity. Macaws did not appear to make dietary selections based on mineral content.

**Conclusions/Significance:**

Parrots represent a remarkable example of a generalist herbivore that consumes seeds destructively despite plant chemical defenses. With the ability to eat toxic foods, rainforest-dwelling parrots exploited a diversity of nutritious foods, even in the dry season when food was scarce for other frugivores and granivores.

## Introduction

The ecological role of parrots in tropical forests may yet be underestimated, but a growing number of recent studies have described the feeding ecology and diets of wild parrots (in the neotropics alone: [1]–[17]). Although often classified as frugivores, most parrots eat seeds in various stages of ripeness as the primary component of their diets, with larger parrots eating a higher proportion of seeds relative to fruit pulp than do smaller parrots [6], [7], [9], [14], [15], [18], [19]. Most of these recent studies classify parrots as pre-dispersal seed predators, regardless of geographic region or habitat type. Only one species of parrot, Pesquet’s Parrot *Psittrichus fulgidus*, is a specialized frugivore, feeding exclusively on the pulp of figs [20]. Although lorikeets consume some fruit pulp along with other vegetative parts, they depend primarily upon nectar and pollen for their energy and nutrition [21]–[22]. When foraging parrots other than lories and Pesquet’s Parrots target fruit, they may consume pulp but at least as often they discard it in favor of the seeds inside [23]. Moreover, parrots eat seeds when the fruits are unripe and therefore before seeds are ready for dispersal [14]. With powerful bills, many parrots appear easily to circumvent the physical protection of hard-shelled seeds [24], but whether seed-borne chemicals can act as deterrents or as poison to these birds has not been well understood. Observations suggest that wild parrots consume seeds that are highly toxic to humans and other vertebrates [25]–[27] but no study to our knowledge has yet explored the relationship between plant chemical defenses and granivory or frugivory by parrots acting as predispersal seed predators. Also as yet unclear is whether these plant foods are nutritionally limiting for wild parrots in lowland humid forests.

The undisturbed lowland Amazonian forest represents the center of biodiversity for Neotropical parrots, hosting up to 25 coexisting species in some places. These forests therefore provide an opportunity to explore the biology of a diverse community of parrots and to evaluate the parrots’ role in this complex ecosystem. Central to this role is that of parrots as predispersal seed predators, able to overcome the chemical defenses of plants while meeting their nutritional requirements. For herbivores, these requirements would center on sufficient protein, lipids, and minerals, while minimizing unusable fiber (i.e., refractory material) and plant secondary compounds. As part of a broader study of parrots in the Peruvian Amazon of Manu National Park [28]–[30], we investigated the foods eaten by 17 species of parrots. In this study, we focus on the species and parts of the plants eaten by parrots, nutritional analysis of foods chosen, and toxicity of the plants included and excluded from the diet. In integrating these behavioral and chemical data with other aspects of the foraging ecology of parrots [28]–[30], our study is the first of which we are aware to evaluate the use of well-defended toxic food by parrots in addition to documenting the nutritional content of the natural foods of adult parrots.

## Methods

### Study Site and Species

We observed foraging in 17 species of parrots at two field sites, Manu National Park (11°57′S; 71°17′ W; hereafter “Manu”) and in the Tambopata-Candamo Reserved Zone (13°10′S; 69°30′W; hereafter “Tambopata”), described in detail elsewhere [28], [30]–[32]. This region lies at the base of the Andes in tropical humid forest in southeastern Perú. These forests have remained remarkably unaffected by modern human activity and retained their pre-Columbian biodiversity at the time of this study. The abundant parrots in the region ranged in size from the diminutive parrotlets (25 g) to the large macaws (>1200 g) and included 8 genera recorded in this study, from large to small (additional description in [29]): *Ara* (*A. ararauna, A. chloropterus, A. macao, A. severus*); *Amazona* (*A. farinosa, A. orchrocephala*)*; Pionus* (*P. menstruus*); *Aratinga* (*A. leucophthalama, A. weddellii*); *Orthopsittaca (O. manilata); Pionites* (*P. leucogaster*); *Pyrilia (P. barrabandi*); *Pyrrhura* (*P. picta, P. rupicola*); *Brotogeris* (*B. cyanoptera, B. sanctithomae*); and *Forpus* (*F. modestus*).

### Foraging Observations

We observed parrots foraging by walking census routes in Manu during the dry seasons (July and August) of 1992 and 1993. We made all Tambopata observations during the wet season (January and February) of 1994. We located foraging birds by direct observation from emergent trees, by hearing vocalizations, or most often, by hearing falling fruit. Observers walked the same pre-established paths through the forest for 1 to 3 hours each day (0630–0930 hours) during these months. We conducted 10–13 morning censuses in each habitat and stratified observations so that each habitat type received equal censusing effort.

When we located foraging parrots, we noted: the species identity of both tree and parrot(s); which plant parts were consumed and the stage of ripeness of fruits and seeds. Because of concern for statistical independence, for this study, we noted only each first observation of parrot species and exploited tree species. Sample size was therefore highly conservative; we noted 224 total unique observations of members of a given species of parrot feeding on a given species of tree. We collected plant samples from the ground below a foraging event, or we climbed the tree and collected fruit directly from the canopy at the same stage of maturity as those being consumed. We defined seed predation as the destructive use of seeds, consumption of whole fruits (such as figs) or of unripe fruits within which seeds might be consumed whole but were not ready for dispersal when consumed. Therefore to estimate granivory (digestion of seeds) in contrast to frugivory (digestion of fruit pulp), we counted as granivory any instance in which we observed whole fruits, unripe fruits, or seeds taken by parrots. We estimated use of unripe fruit or seed based on color and state of plant parts falling to the ground. Plants were identified to species by botanists working in the two reserves [31]–[32] by direct observation of samples or by photographs. Foraging observations as raw data are provided in Dataset S1 (Appendix 1).

### Laboratory Analyses

Following collection, we dissected the plant samples, weighed them fresh, and then dried all samples to constant weight using simple drying ovens at a relatively constant temperature (≤40°C). In laboratories on the UC Davis campus, we re-dried the samples in a vacuum oven overnight (40°C) and ground them for passage through a 1 mm sieve using either a Wiley Mill or a coffee grinder. We determined lipid content by extracting 0.5–1.0 g dried plant material in diethyl ether using a Soxtec extractor, evaporating the ether from the extract, and comparing dried extract to sample proportion by weight [33] **.** We determined crude protein with standard methods using a nitrogen gas analyzer (LECO FP-428, [34]) and multiplied by 6.25 to estimate crude protein as percent of total weight. We measured neutral detergent fiber, which represents the total fiber fraction (cellulose, hemicelluloses and lignin) that make up cell walls within the food tissue, by following Goering & Van Soest [35] with the addition of heat-stable α-amylase [36].

We tested all plant samples for mineral content by digesting 500 mg dry material in 0.5 ml concentrated HNO_3_ and 2 ml 30 percent H_2_O_2_ in a teflon vessel and heated under pressure in a microwave oven (5 min at 40% power, 8 min at 90% power; CEM Corp. MDS 2000). We diluted this to 15 ml with H_2_0, and then analyzed the extracts using inductively coupled plasma optical emission spectroscopy (Thermo Jarrell Ash Atomscan 25) for the following minerals: Na, Mg, P, S, K, Ca, Fe, Zn. We converted raw results to mg/kg using a three point standard curves based on reference solutions for the appropriate element (Fisher).

We tested all plant materials for total phenolic compounds using the Folin-Ciocalteau method [37]. We extracted 300 mg of dried sample in 10 ml of 50 percent methanol at 50°C for 24 hours and tested an aliquot with the Folin-Ciocalteau reagent, reading absorbance at 720 nm. We compared the raw results with a chlorogenic acid standard curve and present the results as percent chlorogenic acid equivalents.

We tested plant materials for toxicity using a standard brine shrimp bioassay [38], [39]. Brine shrimp assays are widely established as proxies for vertebrates in human medicine and agriculture (e.g., [40]–[43]). After extracting 500 mg of dry plant material in 10 ml 100 percent methanol for 24 hours, we pipetted 0 µl, 10 µl, 100 µl, or 500 µl of the extract into three cells of a 12 well tissue culture plate (Falcon) and dried them *in vacuo*. After adding 5 ml of artificial seawater (Instant Ocean, Aquarium Systems) to each cell, we transferred 10 to 25 individual 24-hour old brine shrimp nauplii to each cell. We covered the plates and counted live *versus* moribund or dead shrimp 24 hours later. We analyzed counts using a probit analysis, which estimates the amount of extract that caused 50 percent mortality (LD_50_) using POLO software [44]. To calibrate these brine shrimp LD_50_ values, we tested several pure phenolics, saponins, and other toxins with the following LD_50_ results in mg/g: α-amanitin 0.001, digitonin 0.07, digitoxin 0.02, quercetin >5.0, quinidine 0.32, quinine 0.55, rutin >5, ß-escin 0.05, strychnine 0.11, and tannic acid 0.26.

Determining how nutritional components affect dietary selection in wild animals is notoriously difficult [45]. Rather than testing all plant resources in an attempt to measure “available” *versus* selected plants, we collected samples of plant parts that fit into two broad categories: plants that we had evidence for at least one parrot species consuming (eaten); and parts of those same plants discarded or ignored by foraging parrots (e.g., fruit, seed coats) or similar and abundant reproductive parts of other plants to which parrots had clear access but did not eat (non-eaten). These collections are neither complete, nor are they necessarily representative. Nevertheless, by sampling foods that a given species of parrot does consume and comparing with those that it does not, we can generate a rough approximation of parrot food choices, particularly in macaws where our data are best, and estimate the criteria by which parrots select or reject potential foods. Our results therefore represent an inventory of known food plants for the parrots at this site. Because of our sampling methods, we do not claim to estimate diet or foraging niche of these species.

### Statistical Analyses

To assess how the quality of available foods varied with plant structure, we compared mean values of different plant parts: seeds; whole fruit; fruit pulp; and other parts (nectar, flowers, stems, buds, bark, etc.), using a series of one-way ANOVAs on the various components. For the three largest macaws, we compared plant parts that were eaten by parrots of a given species against plant parts that were available to them but not eaten, using a series of one-way ANOVAs. Where differences among means were significant in one-way ANOVAs, we employed Bonferronni post-hoc analysis for multiple comparisons, including both standard Bonferroni estimates on the 14 univariate ANOVAs, as well as sequential Bonferroni estimates on means of different plant parts within each univariate analysis of components. Although use of Bonferroni or other corrections are appropriate where multiple comparisons are made, a number of researchers question the overly conservative estimates of statistical significance provided by the Bonferroni tests, particularly in disciplines in which data are difficult to come by (such as in this study) [46]–[48]. We therefore discuss all of our results based on the univariate results and present them as hypotheses to be tested by future studies.

## Results

Parrots of the 17 species observed in this study fed from a total of 102 species of plants, for a total of 224 unique observations of parrots of a given species exploiting trees of a given species (Table 1, Table S1, Dataset S1 Appendix 1). Numbers of observations and number of plant species exploited differed among taxa of parrots (Table 1). This variation reflected a combination of conspicuousness of parrots and relative abundance of both parrot and plants, and not degree of specialization on plant species. Most observations in this study were of the large *Ara* macaws, which used 43 percent of the 102 plant species exploited by parrots. Our data herein represent an inventory of tree species exploited by parrots in this region of southern Perú and are not presented as an estimate of diet or niches of the parrot species involved.

**Table 1:** Number of a total of 102 plant species exploited by each of 17 species of parrots in lowland humid forest of Perú, specifying the plant parts used as food.

		Plant part(s)								
Species of parrot	No. ofobs.^1^	Seed(ripe)	Seed(unripe)	Fruit pulp(ripe)	Fruit pulp(unripe)	Wholefruit	Flowers;nectar	Sap	Frond;leaf; stem	Bark;wood
*Ara ararauna*	17	5	3	5	1	1	3	0	2	0
*A. chloropterus*	32	12	10	16	3	0	1	0	1	1
*A. macao*	52	22	12	23	5	2	5	1	0	1
*A. severus*	13	1	2	4	0	0	4	0	1	1
*Amazona farinosa*	11	3	2	4	3	1	2	0	0	0
*A. ochrocephala*	6	1	2	2	0	0	1	0	1	0
*Aratinga leucopthalama*	7	3	0	2	0	1	4	0	0	0
*A. weddellii*	3	1	1	1	0	0	2	0	0	0
*Brotogeris spp.*	14	5	1	6	0	2	5	0	0	0
*B. cyanoptera*	8	0	0	1	0	3	3	0	1	0
*B. sanctithomae*	19	3	0	2	1	7	5	0	4	0
*Orthopsittaca manilata*	1	0	0	1	0	0	0	0	0	0
*Pionites leucogaster*	11	3	2	2	1	1	2	0	1	1
*Pyrrhura picta*	16	1	1	3	0	8	1	0	2	0
*P. rupicola*	7	0	0	2	1	3	1	0	0	0
*Forpus modestus*	3	0	0	0	2	1	0	0	0	0
*Pionus menstruus*	3	1	2	0	0	0	0	0	0	0
*Pyrilia barrabandi*	2	0	1	0	0	0	1	0	0	0

1 Number of observations equals the number of unique observations of individuals of a given species of parrot feeding on some part of a given species of tree, for a total of 224 unique observations of 17 species of parrots collectively exploiting 102 species of trees. Only the first unique combination of parrot and tree species was used to ensure independence of observations (see Methods). Thus the number of observations for a given parrot species is equal to the number of tree species exploited by each species of parrot.

Parrots fed from both reproductive and non-reproductive parts of these plants, but by far most plant species (92%) were exploited for their reproductive parts (Table 1). For these species, we wished to estimate the degree of granivory, that is, seed predation. For 56 percent of the plant species exploited, seeds were consumed directly (Table 1) and in a destructive manner, as indicated by our inspection of debris on the ground below foraging events. Parrots foraged on unripe fruits and seeds of 21 percent of plant species exploited, and all of these were presumably destroyed and not dispersed. For 56 percent of plant species exploited (not necessarily the same species as above), the parrots tore apart ripe and unripe fruits during their foraging activities. Parrots ate the whole fruits only of 15 percent of plant species exploited, providing an estimate of the maximum number of plant species potentially dispersed by parrots. In addition, parrots fed on flower tissue and nectar from an additional 9 percent of plant species, mostly destructively and therefore not as pollen dispersers. Thus for over 80 percent of the food species they exploited, parrots potentially harmed the reproductive capacity of plants during this interaction (Table 1).

Of the plant parts used by parrots, protein and lipid levels were relatively high, as were fiber levels (Table 2, Table 3). Seeds contained significantly more protein and lipid and less fiber than other plant parts (Table 2). Seeds of a variety of species were more than 25 percent crude protein, ranging up to 48 percent protein, and likewise frequently in excess of 30 percent lipid, ranging up to 57 percent lipid (Dataset S1 Appendix 2). Seeds averaged 19 percent protein, compared to 9 percent for whole fruits, and 7 percent for fruit pulp (back-transformed means from Table 2). Similarly, seeds averaged 11 percent lipid, compared to 5 percent for whole fruit and 2 percent for fruit pulp (Table 2). In contrast, seeds averaged 16 percent fiber, compared to 46 percent for whole fruit, 22 percent for fruit pulp, and 40 percent for other plant parts.

**Table 2:** Nutritional content (mean ± SE) of plant species consumed by parrots in lowland humid forests of Perú.^1^

	Seed		Whole fruit		Fruit pulp		Other		Univariate test of significance		
Component	Mean	SE	Mean	SE	Mean	SE	Mean	SE	F	d.f.	P^2^
Crude protein	21.93 a^3^	2.09	9.67 b	1.08	8.05 b	1.17	9.66 b	1.41	12.7	3,64	<0.0000
Fiber (NDF)	24.6a	4.11	48.08 b	3.47	28.08 a,b	5.79	42.76 b	6.07	7.8	3,60	0.0002
Crude fat	22.86 a	3.57	9.87 a	3.67	3.91 b	1.32	2.12 a,b	0.87	4.8	3,57	0.0048
Ash	0.85	0.3	1.85	0.22	1.00	0.336	0.95	0.25	1.6	3,73	0.21
Total phenolics	5.03 a	1.69	2.77 a	0.711	8.27 a	5.24	14.5 b	3.02	5.2	3,67	0.0027
Toxcity LD_50_	1.48	0.327	0.661	0.164	1.27	0.343	2.84	0.62	1.7	3,73	0.1722
Calcium	3050 a	442	853 b	1729	1690 c	337	12500 b	4280	12.5	3,73	<0.0000
Iron	62.2	6.6	80.7	13	57.5	8.04	55.1	9.48	0.5	3,73	0.6672
Potassium	14400	1340	20800	2950	22800	879	14100	2810	2	3,73	0.1288
Magnesium	3380 a	440	3120 a	395	1701 b	358	2490 a	517	4	3,73	0.0109
Sodium	31.8	5.51	36.4	6.3	59.8	9.29	32.9	10.7	0.7	3,73	0.5479
Phosphorus	4990 a	612	255 a,b	262	2980 b	1090	1650 b	337	7.7	3,73	0.0001
Sulphur	3160 a	724	1070 b	86.9	1144 b	424	1930 a,b	601	8.2	3,73	0.0001
Zinc	37.8 a	4.71	16.2 b	1.34	15.5 b	2.7	21.5 a,b	4.1	5.4	3,73	0.0021
Sample size	29		14		14		11				

1 All values are on a dry-weight basis. Units are: percentage for proximate nutritional components; percentage chlorogenic acid equivalents for phenolics; mg/g for estimates of LD_50_; and mg/kg for minerals. All data was log(10) transformed for analysis, and non-transformed values are presented.

2 Using the standard Bonferroni correction for P-values, the following variables retain a statistically significant effect: Crude protein, P<0.00014; calcium, P<0.00014; sulfur, P = 0.0014; phosphorus, P = 0.0014; fiber (NDF), P = 0.0028; zinc, P = 0.0294; total phenolics, P = 0.0378. Fat becomes only marginally significant, P = 0.0672.

3 For each variable, a sequential Bonferroni comparison of means of different plant parts is indicated by letters, i.e., a, b, c, to note significant differences between means. These are provided only for variables with significant univariate effects.

**Table 3:** Nutritional content (mean ± SE) of plant species consumed by macaws compared with plant species not eaten.^1,2,3^

	Eaten		Not eaten		ANOVA			
Plant food component	Mean	N	Mean	N	F	d.f.	P	
Crude protein	16.2±1.78	39	10.7±1.43	24	4.5	1,62	0.037	
Fiber (NDF)	27.7±3.18	39	42.2±4.63	20	6.7	1,58	0.012	
Ash	0.8±0.16	28	1.3±.024	19	4.1	1,46	0.049	
Crude fat	15.6±2.91	40	9.8±3.46	18	0.4	1,57	0.531	
Total phenolics	4.9±1.46	41	11.4±3.51	23	4.5	1,63	0.039	
Toxicity LD50	7.5±5.57	39	5.1±1.86	23	2.7	1,61	0.105	
Calicum	4070±734	42	8320±2210	24	3.6	1,65	0.063	
Iron	61.3±4.9	42	63.2±9.03	24	0.1	1,65	0.759	
Potassium	14600±7150	42	26500±2170	24	0.3	1,65	0.571	
Magnesium	3040±368	42	2260±258	24	1.1	1,65	0.289	
Sodium	38.5±4.82	42	37.4±6.9	24	0.2	1,65	0.662	
Phosphorus	3786±463	42	3030±778	24	4.4	1,65	0.041	
Sulfur	2440±593	42	1670±343	24	0.1	1,65	0.744	
Zinc	27.9±308	42	20±2.41	24	1.1	1,65	0.29	

1 Observations are pooled for all three macaw species, *Ara macao*, *A. chloroptera*, and *A. ararauana*, comparing foods eaten by a member of at least one species of *Ara* macaw with foods not eaten by macaws in this study, but available simultaneously to macaws during their foraging activities.

2 All values are on a dry-weight basis. Units are: percentage for proximate nutritional components; percentage chlorogenic acid equivalents for phenolics; mg/g for estimates of LD_50_; and mg/kg for minerals. All data was log(10) transformed for analysis, and non- transformed values are presented.

3 A straight Bonferroni correction for 14 comparisons yields none of these univariate comparisons statistically significant. We present these data nevertheless, because of criticism of this correction being overly conservative and resulting in excessive Type II error (see Methods), particularly in disciplines such as ecology and behavior where data are difficult to obtain.

Phenolics content of plant foods varied from essentially none to more than 75 percent chlorogenic acid equivalents (Dataset S1 Appendix 2). Mean phenolics content of the different plant parts was highest in the “other” category containing flowers and vegetative plant structures (9 percent), and lowest for seeds and whole fruits (2 percent for each) (Table 2). General toxicity as LD_50_, which we assayed with brine shrimp and calibrated against pure known standards of pure phenolics, saponins, and other toxins (Methods), was frequently high (Dataset S1 Appendix 2). LD_50_ values closely tracked those of phenolics in all plant parts. Many of the plants tested in this study are likely to be highly toxic, particularly those with LD_50_ values of ≤1 mg/g (Table 2).

These plant foods were relatively rich in most macro-minerals (Table 2). For most minerals, plant parts did not different significantly in amounts relative to one another (Table 2). Of those showing significant differences (Table 2), only phosporus, sulphur and zinc tended to be higher in seeds than in other plant parts. Sodium, a mineral limiting to many herbivores, did not differ among plant parts (Table 2).

With relatively larger sample sizes of the *Ara* macaws, we investigated whether these macaws selected foods based upon their nutritional or toxic content. Macaws ate foods that were significantly higher in protein and lower in fiber and phenolics than equivalent plant parts that were available at the time macaws were observed foraging (Table 3). Macaws also ate foods that were higher in fat and lower in measured levels of toxicity, but these comparisons were not statistically significant, unless we consider a one-tailed test for estimates of LD_50_ (Table 3). No major patterns emerged in the mineral content of foods eaten and not eaten by macaws. Calcium levels were marginally lower and phosphorus significantly higher in foods eaten compared to those not eaten (Table 3). Level of sodium was not different in foods eaten compared to those not eaten (Table 3).

## Discussion

Parrots in this study ate a variety of plant species, a pattern common in granivorous parrots living in tropical humid forests worldwide [8]–[9], [11], [15] [49]–[52]. Because our study was an inventory of tree species exploited, our results demonstrated the minimum number of tree species included in the diets of the communities of generalist granivore parrots in lowland Amazonian Perú. This study was not designed to estimate the actual diet of any species. Although we sampled different seasons, our sample sizes were not sufficient to explore nutritional patterns in seasonal use of tree resources. Because no study has yet presented nutritional and toxicity data on foods of free-ranging wild parrots, we present these data in the spirit of making them available for future researchers who will be increasing sample sizes. To this end, the raw data from this study are available in Dataset S1 Appendices 1 and 2.

### Nutrition and Toxicity of Foods

The dietary diversity observed in this study prompts discussion of whether the parrots are seeking nutrients, or avoiding chemicals, or both, by being generalist herbivores and acquiring their food from many different species of plants. We obtained the expected result that seeds are higher in protein and lipids and lower in fiber (refractory material) than is fruit pulp from the same species of plants. Likewise, seeds and fruit analyzed in this study were much higher in protein and lipids and much lower in fiber than were vegetative parts of the same plants. Thus our study corroborated the general findings of studies on digestive physiology reviewed in Karasov & Martinez del Rio [53], but it is the first study to do nutritional analysis on the actual food of adult parrots in the wild. In our study, macaws ate foods that were more nutritious and less well defended with fiber and toxins than foods that were available to them but that they were not observed to eat. These results, combined with the overall picture of granivory in parrots, provide evidence that parrots choose foods based on their nutritional benefits and costs of plant defenses, a behavior demonstrated in other vertebrate herbivores [54]–[58]. As to be expected, the levels of protein and fat in foods eaten by adults in this study are lower than found in the crops wild macaw chicks [59].

In our study, virtually all plant parts chosen by parrots contained phenolics and other substances known to be toxic to vertebrates; a significant proportion of their observed foods contained levels that are considered to be highly toxic. Although direct testing of toxicity of each of these food items in a bird or even a vertebrate was beyond the scope of this study, as well as ethically problematic, the brine-shrimp bioassay was useful in providing a rough measure of toxicity, partly because the toxicities of these parrot foods varied over three orders of magnitude (*cf.* [39]). Indeed, the brine shrimp assay is widely used and accepted in human medicine and agriculture, as a quick and inexpensive proxy for toxicity experienced by vertebrates (see Methods). Accordingly, from comparison with toxicities of well-known plant secondary compounds tested with the same assay, and because some of these seeds, e.g., *Hura* [60] and *Hevea* [61], are known to be poisonous to vertebrates, we conclude that many of the plants consumed by parrots are toxic to vertebrates in general. Secondary compounds are well known to be produced by plants at often high cost to their own growth and reproduction (e.g., [62]) to deter herbivores.

Plant secondary compounds are known to deter foraging in herbivorous birds and mammals, yet toxic foods were nevertheless consumed by these herbivores, presumably as a trade-off in obtaining higher nutrition or abundant available food [54], [57]. Parrots apparently try to avoid toxic food, but our study shows that their foods nonetheless contain measurable levels of toxins and thus avoidence of these compounds is lower priority than choosing foods with higher nutritional content. In other words, parrots are able to overlook the presence of toxins in their choice of nutritious foods. This ability separates parrots from many other avian herbivores targeting fruits of rainforest plants. Many if not most avian frugivores are unable to process toxic food and serve instead as animal dispersal agents and mutualistic partners by digesting only the fruit pulp [63].

No one has yet studied the physiological effects of toxins or costs of detoxification in parrots. Studies of other herbivores reveal that these costs may be high, depending on the environmental demands. For example, processing of secondary plant compounds that serve as toxic deterrents to vertebrate herbivores may have significant effects on sodium and water balance or require high energy or other costs in producing enzymes or carrier proteins such as P-glycoprotein [64]–[66]. Although we do not yet know of specific physiological adaptations of parrots to detoxify their food, consuming clay has been shown experimentally to function *in vivo* in food detoxification in parrots [30]. The variety of foods consumed by parrots in this study may be related to obtaining protein and lipids from toxic foods, supporting the hypothesis that herbivores should increase their dietary diversity when confronting a variety of chemically defended foods [67]. An alternate but not mutually exclusive view is that plant secondary compounds provide herbivores with potential benefits, such as interacting with other molecules to cause foods to be more nutritious or to kill internal parasites [68].

Our study was inconclusive on whether minerals are potentially limiting in the diet and whether parrots chose foods based upon their mineral content. Only one mineral, phosphorus, was higher in foods eaten than not eaten by macaws. Certainly, natural foods of these parrots contain measurable amounts of eight nutritionally important minerals; future studies should compare these levels with those in formulated captive diets. Others have argued that consumption of clay may play a role in mineral acquisition [69]–[71], but our data show that parrots do not select foods based upon content of most potentially limiting minerals, including sodium and calcium.

Understanding and providing adequate nutrition may be critical to recovery programs for endangered and threatened species of granivorous parrots, as it has been for the folivorous Kakapo (*Strigops habroptilus)* [72]. Our data may aid such efforts by allowing comparison of nutrient and mineral contents of their natural foods with those presented to parrots in captivity.

### Mesoscale Predispersal Seed Predators in Tropical Forest Ecosystems

Recent studies of parrots foraging in tropical humid forests stress the high diversity of plant species in the diet, seasonality or unpredictability of fruit production, and the low density and high dispersion of individual trees of any given species typical of lowland forests ([9], [12], [15], [49]–[50], [73]–[75]. These factors combine to present particular challenges to foraging herbivores in tropical rainforests, faced with finding sufficient food of sufficient nutritional quality at all times. Parrots and other forest herbivores commonly meet this challenge with high mobility [29], [76]–[78], and high degree of sociality, which permits the sharing of information on resource availability [11], [29], [79]–[81]. Our study is the first to establish the extent to which toxic foods are routinely included in the diet of granivorous parrots and therefore to highlight how this ability may allow parrots to exploit the mesoscale forager niche so successfully and to avoid competition from other vertebrate herbivores [75].

Our study corroborates that parrots act as predispersal seed predators, as found by numerous other studies of granivorous parrots [4]–[6], [10], [13]–[14], [16], [25] and references therein). In seeking seeds for their high-value nutrition, parrots are clearly not going to serve as dispersal agents for most of these seeds, even though some dispersal may be incidentally accomplished as seed predators forage on their food [82]. Parrots bypass mechanical defenses with their formidable beaks, fortified skulls, and jaw muscles unique to the Psittaciformes [24]. Our study demonstrates in turn just how ineffectual are the chemical defenses that plants mount against parrot herbivory.

Thus parrots as social and mobile seed predators may play a significant role in the structure of topical forest ecosystems. The widespread fragmentation of tropical forests, however, is most likely to have an impact on species that function on such broad spatial scales [63], [83]. Severe habitat destruction and fragmentation in the tropical forests puts parrots, as mesoscale seed predators, at particular risk and may well have contributed to the decline in populations of many species in this order [84].

## Supporting Information

Table S1 **List of 102 species of trees exploited by the community of parrots in Manu National Park and Tambopata Reserve in the lowland humid forest of Perú, showing the number of species of parrot in each genus observed to eat some part of a given species of tree.**
(DOC)

Dataset S1 **Plants eaten by parrots in southeastern Peru, noting part consumed and stage of maturity.**
(XLS)
