# Supplementary material for: Parrots Eat Nutritious Foods despite Toxins
Source: PLoS One. 2012 Jun 5;7(6):e38293. doi: 10.1371/journal.pone.0038293 (PMC3367951; doi:10.1371/journal.pone.0038293)
Supplement: Table S1 — List of 102 species of trees exploited by the community of parrots in Manu National Park and Tambopata Reserve in the lowland humid forest of Perú, showing the number of species of parrot in each genus observed to eat some part of a given species of tree. (DOC) [file pone.0038293.s001.doc]

Table S1. List of 102 species of trees exploited by the community of parrots in Manu National Park and Tambopata Reserve in the lowland humid forest of Perú, showing the number of species of parrot in each genus observed to eat some part of a given species of tree.

______________________________________________________________________________

|  | All parrot species (17 spp.) | *Ara* (4 spp.) | *Amazona* (2 spp.) | *Aratinga* (2 spp.) | *Brotogeris* (2 spp.) | *Orthopsttaca* (1 sp.) | *Pionites* (1 sp.) | *Pyrrhura* (2 spp.) | *Forpus* (1 sp.) | *Pionus* (1 sp.) | *Pyrilia* (1 sp.) |
| --- | --- | --- | --- | --- | --- | --- | --- | --- | --- | --- | --- |
| ______________________________________________________________________________ | | | | | | | | | | | |
| Anacardiacae |  |  |  |  |  |  |  |  |  |  |  |
| *Spondias mombin* | 3 | 2 | 1 |  |  |  |  |  |  |  |  |
| Annonaceae |  |  |  |  |  |  |  |  |  |  |  |
| *Annona* spp. | 2 | 2 |  |  |  |  |  |  |  |  |  |
| *Guateria acutissima* | 1 |  |  |  | 1 |  |  |  |  |  |  |
| *Malmea diclina* | 1 | 1 |  |  |  |  |  |  |  |  |  |
| Apocynaceae |  |  |  |  |  |  |  |  |  |  |  |
| Unid spp*.* | 2 |  |  |  | 2 |  |  |  |  |  |  |
| Bombacacae |  |  |  |  |  |  |  |  |  |  |  |
| *Ceiba pentandra* | 4 | 3 |  |  | 1 |  |  |  |  |  |  |
| *Ceiba samauma* | 2 |  |  | 1 | 1 |  |  |  |  |  |  |
| *Chorisia insignis* | 1 |  |  |  | 1 |  |  |  |  |  |  |
| *Ochroma pyrmidale* | 2 |  |  |  | 2 |  |  |  |  |  |  |
| *Pseudobombax septematum* | 2 |  |  |  | 2 |  |  |  |  |  |  |
| *Quararibea cordata* | 13 | 4 | 3 | 1 | 2 |  | 1 | 1 |  |  | 1 |
| *Quararibea rhombifolia* | 2 | 1 |  |  | 1 |  |  |  |  |  |  |
| Caricacacae |  |  |  |  |  |  |  |  |  |  |  |
| *Carica papaya* | 1 |  |  |  | 1 |  |  |  |  |  |  |
| *Jacaratia digitata* | 1 |  | 1 |  |  |  |  |  |  |  |  |
| Caryocaraceae |  |  |  |  |  |  |  |  |  |  |  |
| *Caryocar spp.* | 2 | 2 |  |  |  |  |  |  |  |  |  |
| Chrysobalanaceae |  |  |  |  |  |  |  |  |  |  |  |
| *Licania britteniana* | 3 |  |  |  | 1 |  |  | 1 | 1 |  |  |
| Combretaceae |  |  |  |  |  |  |  |  |  |  |  |
| *Buchenavia grandis* | 1 | 1 |  |  |  |  |  |  |  |  |  |
| *Combretum assimile* |  | 2 |  |  | 1 |  | 1 | 1 |  |  |  |
| *Terminalia oblonga* |  | 3 |  |  |  |  | 1 |  |  | 1 |  |
| Cucurbitaceae |  |  |  |  |  |  |  |  |  |  |  |
| *Fevillea peruviana* | 1 | 1 |  |  |  |  |  |  |  |  |  |
| Euphorbiaceae |  |  |  |  |  |  |  |  |  |  |  |
| *Croton tessmannii* | 1 | 1 |  |  |  |  |  |  |  |  |  |
| *Hevea guianensis* | 1 | 1 |  |  |  |  |  |  |  |  |  |
| *Hura crepitans* | 2 | 2 |  |  |  |  |  |  |  |  |  |
| *Sapium aereum* | 3 | 2 |  |  | 1 |  |  |  |  |  |  |
| *Sapium marmieri* | 4 | 2 |  |  |  |  | 1 | 1 |  |  |  |
| Unid sp. 1 | 1 |  |  |  | 1 |  |  |  |  |  |  |
| Unid sp. 2 | 2 | 2 |  |  |  |  |  |  |  |  |  |
| Guttiferae |  |  |  |  |  |  |  |  |  |  |  |
| *Rheedia acuminata* | 1 | 1 |  |  |  |  |  |  |  |  |  |
| *Rheedia* sp. | 1 | 1 |  |  |  |  |  |  |  |  |  |
| Lauraceae |  |  |  |  |  |  |  |  |  |  |  |
| Unid sp. | 2 |  |  | 1 |  |  |  | 1 |  |  |  |
| Lecythidaceae |  |  |  |  |  |  |  |  |  |  |  |
| *Couratari guianensis* | 1 | 1 |  |  |  |  |  |  |  |  |  |
| Unid sp. | 1 |  | 1 |  |  |  |  |  |  |  |  |
| Leguminosae |  |  |  |  |  |  |  |  |  |  |  |
| *Bauhina sp.* | 1 | 1 |  |  |  |  |  |  |  |  |  |
| *Cederlinga cateniiformis* | 1 | 1 |  |  |  |  |  |  |  |  |  |
| *Dipteryx alata* | 2 | 2 |  |  |  |  |  |  |  |  |  |
| *Enterolobium cyclocarpum* | 2 | 1 |  |  | 1 |  |  |  |  |  |  |
| *Enterolobium sp.* | 1 |  |  |  | 1 |  |  |  |  |  |  |
| *Erythrina poeppigiana* | 4 | 1 |  | 2 | 1 |  |  |  |  |  |  |
| *Erythrina ulei* | 6 | 1 | 2 | 2 | 1 |  |  |  |  |  |  |
| *Inga edulis* | 2 | 2 |  |  |  |  |  |  |  |  |  |
| *Inga* sp. | 4 | 2 | 1 |  |  |  |  |  |  | 1 |  |
| *Inga* sp. 9421 | 2 | 2 |  |  |  |  |  |  |  |  |  |
| *Parkia* spp. | 1 | 1 |  |  |  |  |  |  |  |  |  |
| *Schizolobium parahybum* | 2 | 2 |  |  |  |  |  |  |  |  |  |
| *Schwartzia* spp. | 3 | 3 |  |  |  |  |  |  |  |  |  |
| Unid sp. | 2 |  | 1 |  |  |  |  |  |  | 1 |  |
| Meliaceae |  |  |  |  |  |  |  |  |  |  |  |
| *Cedrela odorata* | 4 | 3 |  |  |  |  |  | 1 |  |  |  |
| Menispermaceae |  |  |  |  |  |  |  |  |  |  |  |
| *Anomospermum cholran.* | 1 | 1 |  |  |  |  |  |  |  |  |  |
| *A. grandifolium* | 1 | 1 |  |  |  |  |  |  |  |  |  |
| *Borismene japurensis* | 2 | 2 |  |  |  |  |  |  |  |  |  |
| Monimiacea |  |  |  |  |  |  |  |  |  |  |  |
| *Mollineria* sp. | 1 | 1 |  |  |  |  |  |  |  |  |  |
| Moraceae |  |  |  |  |  |  |  |  |  |  |  |
| *Brosimum lactesens* | 6 | 2 | 1 | 1 | 1 |  | 1 |  |  |  |  |
| *Cecropia* sp. | 3 | 1 |  | 1 |  |  |  |  | 1 |  |  |
| *Clarisia racemosa* | 2 | 1 |  |  | 1 |  |  |  |  |  |  |
| *Coussapoa* sp. | 2 |  |  |  |  |  |  | 1 | 1 |  |  |
| *Coussapoa* sp. 1 | 3 |  |  |  | 2 |  |  | 1 |  |  |  |
| *Coussapoa* sp. 2 | 2 |  |  |  |  |  |  | 2 |  |  |  |
| *Coussapoa* sp. 3 | 2 |  |  |  |  |  | 1 | 1 |  |  |  |
| *Ficus carapiensis* | 1 |  |  |  |  |  |  | 1 |  |  |  |
| *Ficus erythrosticta* | 2 |  |  |  | 2 |  |  |  |  |  |  |
| *Ficus insipida* | 2 | 1 |  |  |  |  |  | 1 |  |  |  |
| *Ficus killipii* | 6 | 2 | 1 |  | 2 |  |  | 1 |  |  |  |
| *Ficus mathewsii* | 1 |  |  |  | 1 |  |  |  |  |  |  |
| *Ficus maximus* | 1 |  |  |  |  |  |  | 1 |  |  |  |
| *Ficus perforata* | 5 | 2 |  |  | 2 |  |  | 1 |  |  |  |
| *Ficus pseudokillipii* | 1 |  |  |  | 1 |  |  |  |  |  |  |
| *Ficus* sp. 1 | 1 |  |  |  | 1 |  |  |  |  |  |  |
| *Ficus* sp. 2 | 2 |  |  |  |  |  |  | 2 |  |  |  |
| *Ficus trigona* | 2 |  |  | 1 | 1 |  |  |  |  |  |  |
| *Pseudolmedia laevis* | 6 | 1 | 1 |  | 1 |  | 1 | 1 |  |  | 1 |
| *Sorocea pileata* | 4 | 3 | 1 |  |  |  |  |  |  |  |  |
| Myristicaceae |  |  |  |  |  |  |  |  |  |  |  |
| *Otoba* spp. | 5 | 4 | 1 |  |  |  |  |  |  |  |  |
| *Virola calophylla* | 3 | 2 |  |  |  |  | 1 |  |  |  |  |
| *Virola* sp. | 1 |  |  |  |  |  | 1 |  |  |  |  |
| Palmae |  |  |  |  |  |  |  |  |  |  |  |
| *Astrocaryum murumuru* | 1 | 1 |  |  |  |  |  |  |  |  |  |
| *Iriartea deltoidea* | 4 | 2 |  |  |  |  |  | 2 |  |  |  |
| *Mauritia flexuosa* | 3 | 2 |  |  |  | 1 |  |  |  |  |  |
| *Sheelea phalerata* | 2 | 2 |  |  |  |  |  |  |  |  |  |
| *Socratea exorhiza* | 1 | 1 |  |  |  |  |  |  |  |  |  |
| Papilonaceae |  |  |  |  |  |  |  |  |  |  |  |
| *Centrolobium paraense* | 2 | 2 |  |  |  |  |  |  |  |  |  |
| Rubiaceae |  |  |  |  |  |  |  |  |  |  |  |
| *Calycophyllum spruceanum* | 2 | 1 |  |  |  |  | 1 |  |  |  |  |
| Salicaceae |  |  |  |  |  |  |  |  |  |  |  |
| *Salix* sp. | 2 |  | 1 |  | 1 |  |  |  |  |  |  |
| Sapindaceae |  |  |  |  |  |  |  |  |  |  |  |
| *Cupania cinerea* | 2 | 2 |  |  |  |  |  |  |  |  |  |
| Sapindaceae |  |  |  |  |  |  |  |  |  |  |  |
| *Paullinia* sp. | 1 | 1 |  |  |  |  |  |  |  |  |  |
| Sapotaceae |  |  |  |  |  |  |  |  |  |  |  |
| *Pouteria* sp. | 1 | 1 |  |  |  |  |  |  |  |  |  |
| *Pouteria ulei* | 2 | 2 |  |  |  |  |  |  |  |  |  |
| Staphyleaceae |  |  |  |  |  |  |  |  |  |  |  |
| *Huertea glandulosa* | 1 |  |  |  |  |  |  | 1 |  |  |  |
| Sterculiaceae |  |  |  |  |  |  |  |  |  |  |  |
| *Byttneria asterotricha* | 2 | 2 |  |  |  |  |  |  |  |  |  |
| *Byttneria catalpaefolia* | 2 | 2 |  |  |  |  |  |  |  |  |  |
| *Byttneria pescapraeifolia* | 2 | 2 |  |  |  |  |  |  |  |  |  |
| Tiliaceae |  |  |  |  |  |  |  |  |  |  |  |
| *Apeiba membranacea* | 1 | 1 |  |  |  |  |  |  |  |  |  |
| Ulmaceae |  |  |  |  |  |  |  |  |  |  |  |
| *Ampelocera verrucosa* | 1 | 1 |  |  |  |  |  |  |  |  |  |
| *Celtis iguanea* | 1 |  |  |  | 1 |  |  |  |  |  |  |
| Verbenaceae |  |  |  |  |  |  |  |  |  |  |  |
| *Citharexylum poeppigii* | 1 | 1 |  |  |  |  |  |  |  |  |  |
| Violaceae |  |  |  |  |  |  |  |  |  |  |  |
| *Leonia glycicarpa* | 1 | 1 |  |  |  |  |  |  |  |  |  |
| *Rinorea* sp. 9414 | 2 | 2 |  |  |  |  |  |  |  |  |  |
| Vochysiaceae |  |  |  |  |  |  |  |  |  |  |  |
| *Qualea* | 1 | 1 |  |  |  |  |  |  |  |  |  |
| Family unidentified |  |  |  |  |  |  |  |  |  |  |  |
| 1 | 1 |  |  |  |  |  |  | 1 |  |  |  |
| 2 | 1 |  | 1 |  |  |  |  |  |  |  |  |
| 3 | 1 |  |  |  |  |  | 1 |  |  |  |  |
| 4 | 2 | 2 |  |  |  |  |  |  |  |  |  |
| 5 | 2 | 2 |  |  |  |  |  |  |  |  |  |

______________________________________________________________________________
